# Supplementary figures and images for: Genome-wide identification of ABA receptor PYL family and expression analysis of PYLs in response to ABA and osmotic stress in Gossypium
Source: PeerJ. 2017 Dec 6;5:e4126. doi: 10.7717/peerj.4126 (PMC5723141; doi:10.7717/peerj.4126)

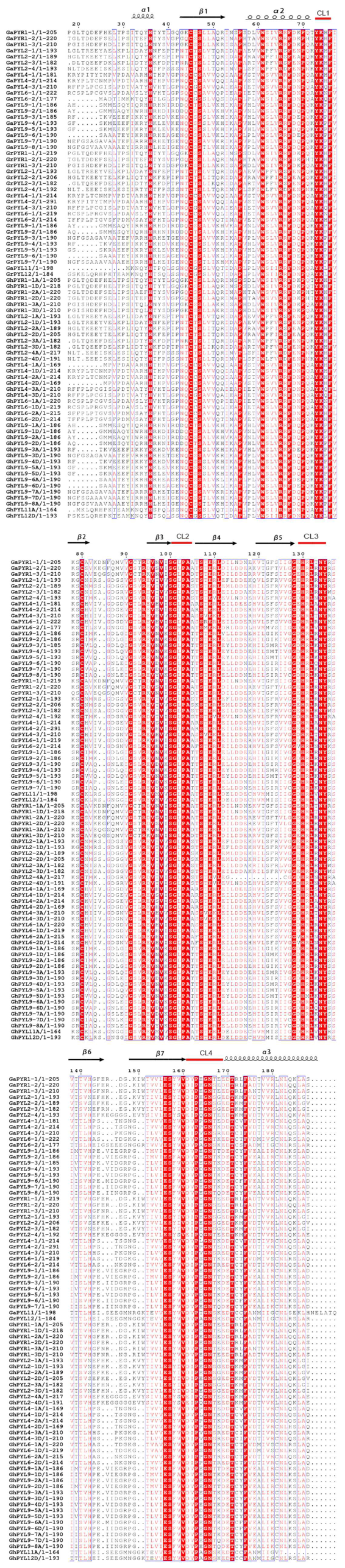

Supplement: Figure S1 [file peerj-05-4126-s001.png]
