# Supplementary material for: Genome-wide identification of ABA receptor PYL family and expression analysis of PYLs in response to ABA and osmotic stress in Gossypium
Source: PeerJ. 2017 Dec 6;5:e4126. doi: 10.7717/peerj.4126 (PMC5723141; doi:10.7717/peerj.4126)
Supplement: Table S1 [file peerj-05-4126-s003.docx]

**Table S1** The gene primers used for quantitative real-time RT-PCR experiments

| Genes | AGI number | NCBI accession | Forward primers (5ʹ-3ʹ) | Reverse primers (5ʹ-3ʹ) |
| --- | --- | --- | --- | --- |
| *GhPYR1-1A*  *GhPYR1-1D*  *GhPYR1-2A*  *GhPYR1-2D*  *GhPYR1-3A*  *GhPYR1-3D*  *GhPYL2-1A*  *GhPYL2-1D*  *GhPYL2-2A*  *GhPYL2-2D*  *GhPYL2-3A*  *GhPYL2-3D*  *GhPYL2-4A*  *GhPYL2-4D*  *GhPYL4-1A*  *GhPYL4-1D*  *GhPYL4-2A*  *GhPYL4-2D*  *GhPYL4-3A*  *GhPYL4-3D*  *GhPYL6-1A*  *GhPYL6-1D*  *GhPYL6-2A*  *GhPYL6-2D*  *GhPYL9-1A*  *GhPYL9-1D*  *GhPYL9-2A*  *GhPYL9-2D*  *GhPYL9-3A*  *GhPYL9-3D*  *GhPYL9-4D*  *GhPYL9-5A*  *GhPYL9-5D*  *GhPYL9-6A*  *GhPYL9-6D*  *GhPYL9-7A*  *GhPYL9-7D*  *GhPYL9-8A*  *GhPYL11A*  *GhPYL12D*  *GhUBQ7* | Gh_A03G0015  Gh_D03G1860  Gh_A11G0270  Gh_D11G0290  Gh_A12G1895  Gh_D12G2076  Gh_A05G0336  Gh_D05G0441  Gh_A08G2221  Gh_D08G2587  Gh_A07G2326  Gh_D07G0193  Gh_A10G0677  Gh_D10G0710  Gh_A01G1990  Gh_D09G1585  Gh_A09G2421  Gh_D01G2250  Gh_A05G2630  Gh_D05G2920  Gh_A10G2142  Gh_D10G2388  Gh_A06G1418  Gh_D06G1764  Gh_A08G1117  Gh_D11G1013  Gh_A11G0870  Gh_D08G1399  Gh_A11G0224  Gh_D04G0019  Gh_D11G0238  Gh_A12G2127  Gh_D12G2306  Gh_A09G1646  Gh_D09G1740  Gh_A05G3585  Gh_D12G2694  Gh_A12G2278  Gh_A05G1297  Gh_D05G1468  Gh_A11G0969 | Pr032826479  Pr032826480  Pr032826481  Pr032826482  Pr032826483  Pr032826484  Pr032826447  Pr032826448  Pr032826449  Pr032826450  Pr032826451  Pr032826452  Pr032826453  Pr032826454  Pr032826455  Pr032826456  Pr032826457  Pr032826458  Pr032826459  Pr032826460  Pr032826461  Pr032826462  Pr032826463  Pr032826464  Pr032826465  Pr032826466  Pr032826467  Pr032826468  Pr032826469  Pr032826470  Pr032826471  Pr032826472  Pr032826473  Pr032826474  Pr032826475  Pr032826476  Pr032826477  Pr032826478  Pr032826445  Pr032826446  Pr032826524 | GGGTTTAGCATCATCGGTGG  GGACTGTTGTTCTGGAATCAT  CTGGACGATGAGCGAGAGGT  TATTCTGGACGATGAGCGAG  CAACTACTCAGTTGGTCCCG  CACCTACTCAGATGGTCCCG  AAGTACGGAAAGGCTGGAGT  GGCTGAGAAACTACAGGTCG  CCCTCACGGCCTAACCA  TTTCTTCATCGTCAACAACAG  TTCATCAAGAGCTGTAACATC  TCGTCAAGAGCTGCAACATCA  CTCAGCACCCAAAGCAACA  ATGACGATGAGCATGTTTTGA  TCATCGGTGGAGAACATCG  CAACTACTCAGTTGGTCCCG  CGTCTGGTCTGTTGTCCGC  GTCATCGGTGGAGAACATCG  ATCCACCTCTCGCCGC  ATCCACCGCTCGCCGC  TCAACCCATCGAAGCCC  CCCAATCGAGACGGCTT  GAGGCTTGAAATCCTTGACGA  CACCGCCGTTGGCACCT  GGGAACACCACGGACGA  CAGCCGCAGAAGTACAAGCC  ATCAGCCGCAAAAGTATAA  AAGGAGATTTGACCAGCCGC  GTGAGGAGATTTGATGAACCC  AGTTTGTAAGGAGACATCATC  CGATCAGCCGCAGAAGTACA  TAAGGAGGTTTGACCAACCC  GAACGGGTTTAGCAAGAGGG  TAGATGGAAGACCTGGGACA  TAAGGATTATCGGTGGGGA  GAGTTTTTAAGGAGACGTCAT  CGGATTCAGTGGTGCGG  GTAGTGTGGCGGCGGTT  AAAACCAGTATCACTCCCCTC  AACCAGTATCACACCTCTCAG  GAAGGCATTCCACCTGACCA | TGATTCCAGAACAACGGTCCA  GAAAAGTACAGGGACTGAAGC  ACAGCCGCGTATCGTCCT  AAACAGCCGTGTGTCTTCC  CCACCACCATTTGCGAA  CCATTTGCGAGCCTTGTT  ATAAGACTCTAAAACGATGGTGTAA  GTAGAGATCCCATTGCCACA  GATGCGTTGGGTTATGAGG  GCGTTGGGTTATGAGGGA  TGACCAGTAGTTCCTCAGCC  CCGCCCACCACCCTAAA  GTTTGAGGATGAAGGAAAGGTA  CTTGAGGTATCTCCACCGAAT  TGAGCAAGTGACTGCAGGT  CCACCACCATTTGCGAA  CCAACTTCACCATCCCCAA  TGAGCCAGTGAGTGCAGGT  GATGGCTTGAACAACTGCTG  GACGAACCACTGACCAAACG  GCCTCCAACCACGCTGA  CCTCCGACCACGCTGAA  TCTCTGGTATTACCGGGCG  GTCGGGAAAGGAGACGCCA  TGATAGGCTGTGTCTGATCCAT  CCTGAGACGGTGGTCGCCT  TAACCTCTGGATGGACCG  CGCCGACGATTTTGATGCC  ATGGACTGTAACAATGGATGAG  GGTTGGTCAAATCGCCT  CTCTAACCTTTCGGTGCTAGTTG  TCAATGACCTCAGGGTGGAC  ACAACACACCTGCTAACAAATGG  CCGTTCAGTCCGGTCCTG  CCGTTCAGTCTGGTCTTGC  GCCTCACCAGAGACCAAACG  TCCTGACTTGACATAAACTTCTCTA  GGGTTTATACTTCTGAGGTTGG  CAACCCTGACACCACCAT  CCACCATCACTTCACGCA  CTTGACCTTCTTCTTCTTGTGCTTG |
