# Supplementary material for: Genome-wide identification of ABA receptor PYL family and expression analysis of PYLs in response to ABA and osmotic stress in Gossypium
Source: PeerJ. 2017 Dec 6;5:e4126. doi: 10.7717/peerj.4126 (PMC5723141; doi:10.7717/peerj.4126)
Supplement: Table S2 [file peerj-05-4126-s004.docx]

**Table S2** The gene primers applied in yeast two-hybrid experiments

| Genes | AGI number | NCBI accession | Forward primers (5ʹ-3ʹ) | Reverse primers (5ʹ-3ʹ) |
| --- | --- | --- | --- | --- |
| GhPYR1-1A  GhPYR1-1D  GhPYR1-2A  GhPYR1-2D  GhPYR1-3A  GhPYL2-1A  GhPYL2-2A  GhPYL2-2D  GhPYL2-3D  GhPYL4-1A  GhPYL4-2A  GhPYL4-2D  GhPYL4-3A  GhPYL6-1D  GhPYL6-2A  GhPYL6-2D  GhPYL9-1A  GhPYL9-2A  GhPYL9-3D  GhPYL9-4D  GhPYL9-5D  GhPYL9-6A  GhPYL9-6D  GhPYL9-7D  GhPYL11A  GhABI1A  GhABI1D | Gh_A03G0015  Gh_D03G1860  Gh_A11G0270  Gh_D11G0290  Gh_A12G1895  Gh_A05G0336  Gh_A08G2221  Gh_D08G2587  Gh_D07G0193  Gh_A01G1990  Gh_A09G2421  Gh_D01G2250  Gh_A05G2630  Gh_D10G2388  Gh_A06G1418  Gh_D06G1764  Gh_A08G1117  Gh_A11G0870  Gh_D04G0019  Gh_D11G0238  Gh_D12G2306  Gh_A09G1646  Gh_D09G1740  Gh_D12G2694  Gh_A05G1297  Gh_A07G0123  Gh_D07G2383 | Pr032826519  Pr032826520  Pr032826521  Pr032826522  Pr032826523  Pr032826500  Pr032826501  Pr032826502  Pr032826503  Pr032826504  Pr032826505  Pr032826506  Pr032826507  Pr032826508  Pr032826509  Pr032826510  Pr032826511  Pr032826512  Pr032826513  Pr032826514  Pr032826515  Pr032826516  Pr032826517  Pr032826518  Pr032826499  Pr032826497  Pr032826498 | CCGGAATTCATGGCTGAACCAGAATCC  CCGGAATTCATGGCTGAACCAGAATCC  CCGGAATTCATGGCAGACCCGAAACCC  CCGGAATTCATGGCAGACCCGAAACCC  CCGGAATTCATGGCAGTCTCAAAACCCGC  CCGGAATTCATGGATAACTCAAGCGAGCAA  CCGGAATTCATGGACTCAGCGGAGCCAC  CCGGAATTCATGGACTCAGCGGAGCCAC  CGCGGATCCATGGACTCGGACCTAACCC  CCGGAATTCATGCCAGTCCCAGACGCC  CCGGAATTCATGCATGCCAATCCTCCAAA  CCGGAATTCATGCATGCCAATCCTCCAAA  CCGGAATTCATGCCTTCCTCACTGAAGCTCCAT  CCGGAATTCATGCCTTCCCCTTTGCAACT  CCGGAATTCATGCCTTCCTCTTTGCAGC  CCGGAATTCATGCCTTCCTCTTTGCAGC  CCGGAATTCATGAACGGTGATGATGCTCAC  CCGGAATTCATGAACGGTGGTGATGCTTA  CCGGAATTCATGAATGGGAATAGCAATGGA  CCGGAATTCATGGTGACCAATAATTATATCACCA  CCGGAATTCATGGTGACCAATGATTATATCACCATG  CCGGAATTCATGAACGTGAGTAGCAGTGC  CCGGAATTCATGAACGTGAGTAGCAGTGC  CCGGAATTCATGAATGGGCATAGCAACGGAT  CCGGAATTCATGAAGCTTCAACGCCATCCC  CCGGAATTCATGATGGAAGAAGTATCTGGT  CCGGAATTCATGATGGAAGAAGTATCTGGT | CGCGGATCCCATCACCTGTGATTTATTACA  CGCGGATCCCATCACCTGTGATTTATTACA  CGCGGATCCCATCACCTGTGGTTTATTACCG  CGCGGATCCCATCACCTGTGGTTTATTACCG  CGCGGATCCTGAATTATTACCGTCATTATCA  CGCGGATCCATCATGTCTCGGTTCATGAAG  CGCGGATCCATCATGTCCATGAACTGAACC  CGCGGATCCATCATGTCCATGAACTGAACC  GACGTCGACATCATGCCCATGAAAAGAACC  CGCGGATCCTTTCCGGCTTGCTAGATT  CGCGGATCCTTTGCGCCTGGAGAGATTCT  CGCGGATCCTTTGCGCCTGGAGAGATTCT  CGCGGATCCTTCTGTTCTAGCCATATTTTCTGC  CGCGGATCCTGGGGATGATGAATATTTTT  CGCGGATCCGGGAGATGATGACAATGAT  CGCGGATCCGGGAGATGATGACAATGAT  CGCGGATCCGTATCGGTTGATAGGCTGTGT  CGCGGATCCGTATCCGTTGATAGGCTCTG  CGCGGATCCAAGATCAATGGGTTCTGTC  CGCGGATCCCATTCTTTCGATCGGCTC  CGCGGATCCCATTCTTTTGATTGGCTCGGT  CGCGGATCCTCTTTCAGCTCCCAACTT  CGCGGATCCTCTTTCAGCTCCCAACTT  CGCGGATCCTAGATCAATGGGTTCAGTCCGG  CGCGGATCCAGTTACACCAATTATCATATTGGC  GCGGAGCTCTGTTTTCTTCTTAAATTTTCTCT  GCGGAGCTCTGTTTTCTTCTTAAATTTTCTCT |
