# Supplementary material for: Genome-wide identification of ABA receptor PYL family and expression analysis of PYLs in response to ABA and osmotic stress in Gossypium
Source: PeerJ. 2017 Dec 6;5:e4126. doi: 10.7717/peerj.4126 (PMC5723141; doi:10.7717/peerj.4126)
Supplement: Table S3 [file peerj-05-4126-s005.docx]

**Table S3** The PYL members in *Gossypium*

| Gene name | ID | PI | MW (kDa) | Hydrop  -hylicity | Predicted subcellular localization | Amino acid residues | Coding sequences |
| --- | --- | --- | --- | --- | --- | --- | --- |
| GaPYL2-1 | Cotton_A_13999 | 5.51 | 21.69 | -0.451 | cytoplasm | 193 | 582 |
| GaPYL2-2 | Cotton_A_11886 | 5.81 | 21.05 | -0.316 | cytoplasm | 189 | 570 |
| GaPYL2-3 | Cotton_A_05614 | 6.21 | 20.58 | -0.375 | cytoplasm | 182 | 549 |
| GaPYL2-4 | Cotton_A_11801 | 5.11 | 21.38 | -0.236 | cytoplasm, nucleus | 193 | 582 |
| GaPYL4-1 | Cotton_A_00296 | 6.28 | 19.87 | -0.109 | cytoplasm | 181 | 546 |
| GaPYL4-2 | Cotton_A_22319 | 7.7 | 23.42 | -0.245 | cytoplasm | 214 | 645 |
| GaPYL4-3 | Cotton_A_31479 | 6.49 | 22.84 | -0.028 | cytoplasm | 210 | 633 |
| GaPYL6-1 | Cotton_A_15835 | 7.29 | 24.19 | -0.295 | cytoplasm | 222 | 669 |
| GaPYL6-2 | Cotton_A_17533 | 5.3 | 19.11 | -0.095 | cytoplasm | 177 | 534 |
| GaPYL9-1 | Cotton_A_24449 | 6.3 | 21.13 | -0.348 | cytoplasm | 186 | 561 |
| GaPYL9-2 | Cotton_A_14394 | 5.8 | 21.92 | -0.307 | cytoplasm | 186 | 561 |
| GaPYL9-3 | Cotton_A_36156 | 7.71 | 21.14 | -0.436 | nucleus | 185 | 558 |
| GaPYL9-4 | Cotton_A_13258 | 5.81 | 21.83 | -0.314 | cytoplasm | 193 | 582 |
| GaPYL9-5 | Cotton_A_23205 | 5.81 | 21.83 | -0.314 | cytoplasm | 193 | 582 |
| GaPYL9-6 | Cotton_A_08084 | 5.89 | 21.16 | -0.268 | cytoplasm | 190 | 573 |
| GaPYL9-7 | Cotton_A_20366 | 5.58 | 21.01 | -0.272 | cytoplasm | 190 | 573 |
| GaPYL9-8 | Cotton_A_17740 | 5.89 | 21.12 | -0.147 | cytoplasm | 190 | 573 |
| GaPYL11 | Cotton_A_11270 | 5.02 | 21.74 | -0.148 | cytoplasm, nucleus | 198 | 597 |
| GaPYR1-1 | Cotton_A_23118 | 5.47 | 22.85 | -0.364 | nucleus | 205 | 618 |
| GaPYR1-2 | Cotton_A_09168 | 5.11 | 24.18 | -0.313 | cytoplasm | 220 | 663 |
| GaPYR1-3 | Cotton_A_07906 | 7.13 | 23.41 | -0.386 | cytoplasm, nucleus | 210 | 633 |
| GrPYL2-1 | 009G045700 | 5.76 | 21.73 | -0.454 | cytoplasm | 193 | 582 |
| GrPYL2-2 | 004G287300 | 6.17 | 22.79 | -0.303 | cytoplasm | 206 | 621 |
| GrPYL2-3 | 001G023000 | 5.74 | 20.36 | -0.321 | cytoplasm | 182 | 549 |
| GrPYL2-4 | 011G081100 | 5.38 | 21.3 | -0.212 | nucleus | 192 | 579 |
| GrPYL4-1 | 006G185000 | 7.16 | 23.36 | -0.2 | cytoplasm | 214 | 645 |
| GrPYL4-2 | 002G266100 | 8.73 | 32.22 | -0.127 | cytoplasm | 291 | 876 |
| GrPYL4-3 | 009G323000 | 6.49 | 22.84 | -0.028 | cytoplasm | 210 | 633 |
| GrPYL6-1 | 011G290300 | 8.23 | 23.82 | -0.284 | cytoplasm | 219 | 660 |
| GrPYL6-2 | 010G194800 | 6.44 | 23.32 | -0.113 | cytoplasm | 214 | 645 |
| GrPYL9-1 | 007G107500 | 6.16 | 20.92 | -0.34 | cytoplasm | 186 | 561 |
| GrPYL9-2 | 004G152300 | 5.89 | 21.15 | -0.391 | cytoplasm | 186 | 561 |
| GrPYL9-3 | 012G003800 | 5.69 | 20.94 | -0.261 | cytoplasm | 190 | 573 |
| GrPYL9-4 | 007G026100 | 7.05 | 22.07 | -0.411 | cytoplasm | 193 | 582 |
| GrPYL9-5 | 008G253700 | 6.6 | 21.82 | -0.374 | nucleus | 193 | 582 |
| GrPYL9-6 | 006G200700 | 5.71 | 21.12 | -0.249 | cytoplasm | 190 | 573 |
| GrPYL9-7 | 008G270200 | 5.97 | 21.12 | -0.184 | cytoplasm | 190 | 573 |
| GrPYL12 | 009G162000 | 7 | 20.33 | -0.291 | cytoplasm | 184 | 555 |
| GrPYR1-1 | 003G181600 | 5.47 | 24.49 | -0.29 | nucleus | 219 | 660 |
| GrPYR1-2 | 007G031800 | 5.05 | 24.18 | -0.378 | cytoplasm, nucleus | 220 | 663 |
| GrPYR1-3 | 008G226500 | 6.12 | 23.26 | -0.382 | cytoplasm, nucleus | 210 | 633 |
| GhPYL2-1A | Gh_A05G0336 | 5.61 | 21.71 | -0.463 | cytoplasm | 193 | 582 |
| GhPYL2-1D | Gh_D05G0441 | 5.76 | 21.74 | -0.453 | cytoplasm | 193 | 582 |
| GhPYL2-2A | Gh_A08G2221 | 5.98 | 21.05 | -0.33 | cytoplasm | 189 | 570 |
| GhPYL2-2D | Gh_D08G2587 | 6.17 | 22.67 | -0.306 | cytoplasm | 205 | 618 |
| GhPYL2-3A | Gh_A07G2326 | 5.5 | 20.48 | -0.345 | cytoplasm | 182 | 549 |
| GhPYL2-3D | Gh_D07G0193 | 5.74 | 20.38 | -0.331 | cytoplasm | 182 | 549 |
| GhPYL2-4A | Gh_A10G0677 | 6.31 | 23.81 | -0.319 | nucleus | 217 | 654 |
| GhPYL2-4D | Gh_D10G0710 | 5.1 | 21.14 | -0.179 | cytoplasm | 191 | 576 |
| GhPYL4-1A | Gh_A01G1990 | 5.94 | 18.3 | -0.065 | cytoplasm | 169 | 510 |
| GhPYL4-1D | Gh_D09G1585 | 7.7 | 23.43 | -0.217 | cytoplasm | 214 | 645 |
| GhPYL4-2A | Gh_A09G2421 | 7.7 | 23.42 | -0.245 | cytoplasm | 214 | 645 |
| GhPYL4-2D | Gh_D01G2250 | 6.02 | 18.34 | -0.033 | cytoplasm | 169 | 510 |
| GhPYL4-3A | Gh_A05G2630 | 6.74 | 210 | -0.03 | cytoplasm | 210 | 633 |
| GhPYL4-3D | Gh_D05G2920 | 6.49 | 22.84 | -0.028 | cytoplasm | 210 | 633 |
| GhPYL6-1A | Gh_A10G2142 | 7.25 | 23.93 | -0.302 | cytoplasm | 220 | 663 |
| GhPYL6-1D | Gh_D10G2388 | 8.23 | 23.82 | -0.284 | cytoplasm | 219 | 660 |
| GhPYL6-2A | Gh_A06G1418 | 6.78 | 23.38 | -0.119 | cytoplasm | 215 | 648 |
| GhPYL6-2D | Gh_D06G1764 | 6.44 | 23.32 | -0.113 | cytoplasm | 214 | 645 |
| GhPYL9-1A | Gh_A08G1117 | 6.13 | 21.16 | -0.39 | cytoplasm | 186 | 561 |
| GhPYL9-1D | Gh_D11G1013 | 5.97 | 20.97 | -0.334 | cytoplasm | 186 | 561 |
| GhPYL9-2A | Gh_A11G0870 | 5.8 | 20.92 | -0.307 | cytoplasm | 186 | 561 |
| GhPYL9-2D | Gh_D08G1399 | 5.89 | 21.15 | -0.391 | cytoplasm | 186 | 561 |
| GhPYL9-3A | Gh_A11G0224 | 6.66 | 22.07 | -0.411 | cytoplasm | 193 | 582 |
| GhPYL9-3D | Gh_D04G0019 | 5.69 | 20.94 | -0.261 | cytoplasm | 190 | 573 |
| GhPYL9-4D | Gh_D11G0238 | 6.71 | 22.05 | -0.405 | cytoplasm | 193 | 582 |
| GhPYL9-5A | Gh_A12G2127 | 5.81 | 21.85 | -0.312 | cytoplasm | 193 | 582 |
| GhPYL9-5D | Gh_D12G2306 | 5.88 | 21.86 | -0.32 | cytoplasm | 193 | 582 |
| GhPYL9-6A | Gh_A09G1646 | 5.89 | 21.16 | -0.268 | cytoplasm | 190 | 573 |
| GhPYL9-6D | Gh_D09G1740 | 5.71 | 21.12 | -0.249 | cytoplasm | 190 | 573 |
| GhPYL9-7A | Gh_A05G3585 | 5.76 | 20.94 | -0.255 | cytoplasm | 190 | 573 |
| GhPYL9-7D | Gh_D12G2694 | 6.23 | 21.09 | -0.162 | cytoplasm | 190 | 573 |
| GhPYL9-8A | Gh_A12G2278 | 5.89 | 21.12 | -0.147 | cytoplasm | 190 | 573 |
| GhPYL11A | Gh_A05G1297 | 5.94 | 18.02 | -0.248 | cytoplasm | 164 | 495 |
| GhPYL12D | Gh_D05G1468 | 7 | 21.49 | -0.28 | cytoplasm | 193 | 582 |
| GhPYR1-1A | Gh_A03G0015 | 5.47 | 22.85 | -0.364 | nucleus | 205 | 618 |
| GhPYR1-1D | Gh_D03G1860 | 5.34 | 24.41 | -0.324 | nucleus | 218 | 657 |
| GhPYR1-2A | Gh_A11G0270 | 5.11 | 24.08 | -0.343 | cytoplasm, nucleus | 220 | 663 |
| GhPYR1-2D | Gh_D11G0290 | 5.05 | 24.18 | -0.378 | cytoplasm, nucleus | 220 | 663 |
| GhPYR1-3A | Gh_A12G1895 | 6.75 | 23.31 | -0.367 | cytoplasm | 210 | 633 |
| GhPYR1-3D | Gh_D12G2076 | 6.29 | 23.32 | -0.39 | cytoplasm | 210 | 633 |
| GbPYL2-1A | AA31030 | 5.61 | 21.71 | -0.463 | cytoplasm | 193 | 582 |
| GbPYL2-1D | DD16128 | 5.62 | 21.8 | -0.48 | cytoplasm | 193 | 582 |
| GbPYL2-2A | AA34547 | 5.81 | 21.02 | -0.313 | cytoplasm | 189 | 570 |
| GbPYL2-2D | DD10391 | 6.17 | 21.05 | -0.33 | cytoplasm | 189 | 570 |
| GbPYL2-2Dʹ | DD38339 | 6.17 | 21.05 | -0.33 | cytoplasm | 189 | 570 |
| GbPYL2-3A | AA28868 | 5.3 | 28.4 | -0.29 | cytoplasm | 255 | 768 |
| GbPYL2-3D | DD32447 | 5.4 | 21.08 | -0.138 | cytoplasm | 188 | 567 |
| GbPYL2-4A | AA22659 | 5.23 | 21.22 | -0.22 | cytoplasm | 191 | 576 |
| GbPYL2-4D | DD23620 | 5.1 | 21.12 | -0.152 | nucleus | 190 | 573 |
| GbPYL4-1A | AA24072 | 7.71 | 28.68 | -0.132 | cytoplasm | 259 | 780 |
| GbPYL4-1D | DD31762 | 8.2 | 23.49 | -0.23 | cytoplasm | 214 | 645 |
| GbPYL4-2A | AA31755 | 7.7 | 23.43 | -0.22 | cytoplasm | 214 | 645 |
| GbPYL4-3A | AA01168 | 6.49 | 22.85 | -0.028 | cytoplasm | 210 | 633 |
| GbPYL4-3D | DD09701 | 6.49 | 22.85 | -0.028 | cytoplasm | 210 | 633 |
| GbPYL6-1A | AA18332 | 7.25 | 23.93 | -0.302 | cytoplasm | 220 | 663 |
| GbPYL6-1D | DD29255 | 8.23 | 23.82 | -0.284 | cytoplasm | 219 | 660 |
| GbPYL6-2A | AA13391 | 6.62 | 23.29 | -0.108 | cytoplasm | 214 | 645 |
| GbPYL6-2D | DD20934 | 6.41 | 38.93 | -0.08 | cytoplasm | 355 | 1068 |
| GbPYL6-2Aʹ | AA15331 | 5.3 | 19.11 | -0.095 | cytoplasm | 177 | 534 |
| GbPYL6-2Dʹ | DD25577 | 4.73 | 12.64 | -0.109 | nucleus | 116 | 351 |
| GbPYL9-1D | DD37081 | 5.8 | 20.92 | -0.307 | cytoplasm | 186 | 561 |
| GbPYL9-8A | AA28339 | 6.7 | 17.36 | -0.173 | cytoplasm | 158 | 477 |
| GbPYL9-7A | AA19258 | 9.51 | 15.32 | -0.289 | cytoplasm | 139 | 420 |
| GbPYL9-2D | DD28661 | 5.76 | 22.28 | -0.42 | cytoplasm | 196 | 591 |
| GbPYL9-3A | AA40052 | 6.71 | 22.05 | -0.405 | cytoplasm | 193 | 582 |
| GbPYL9-3D | DD31179 | 5.69 | 20.94 | -0.261 | cytoplasm | 190 | 573 |
| GbPYL9-4D | DD15345 | 6.71 | 24.26 | -0.353 | cytoplasm | 212 | 639 |
| GbPYL9-5A | AA38557 | 5.81 | 21.85 | -0.312 | cytoplasm | 193 | 582 |
| GbPYL9-5D | DD26619 | 5.85 | 17.96 | -0.389 | cytoplasm | 159 | 480 |
| GbPYL9-6A | AA22571 | 5.74 | 24.1 | -0.333 | cytoplasm | 218 | 657 |
| GbPYL9-6D | DD17050 | 5.71 | 21.12 | -0.249 | cytoplasm | 190 | 573 |
| GbPYL9-6Dʹ | DD32170 | 5.89 | 21.15 | -0.255 | cytoplasm | 190 | 573 |
| GbPYL9-7D | DD31997 | 5.89 | 21.09 | -0.159 | cytoplasm | 190 | 573 |
| GbPYL11A | AA37880 | 6.2 | 18.12 | -0.267 | cytoplasm | 164 | 495 |
| GbPYL12D | DD17498 | 6.2 | 18.61 | -0.164 | cytoplasm | 169 | 510 |
| GbPYR1-1D | DD23506 | 5.47 | 22.87 | -0.364 | nucleus | 205 | 618 |
| GbPYR1-2D | DD21981 | 5.13 | 24.18 | -0.378 | cytoplasm, nucleus | 220 | 663 |
| GbPYR1-3A | AA29004 | 7.13 | 23.31 | -0.367 | cytoplasm | 210 | 633 |
| GbPYR1-3D | DD35789 | 5.85 | 20.67 | -0.43 | cytoplasm | 186 | 561 |

ID: gene identifier; PI: isoelectric point; MW: molecular weight.
